# Methods for joint genetic prediction of multiple ordinal categorical and continuous traits

**DOI:** 10.1093/genetics/iyag086

**Published:** 2026-03-26

**Authors:** Matias Bermann, Andres Legarra, Ignacy Misztal, Daniela Lourenco

**Affiliations:** Department of Animal and Dairy Science, University of Georgia, Athens, GA 30602, United States; Department of Animal and Dairy Science, University of Georgia, Athens, GA 30602, United States; Council on Dairy Cattle Breeding, Bowie, MD 20716, United States; Department of Animal and Dairy Science, University of Georgia, Athens, GA 30602, United States; Department of Animal and Dairy Science, University of Georgia, Athens, GA 30602, United States

**Keywords:** threshold models, liability, binary traits, expectation-maximization, Newton-Raphson, single-step GBLUP

## Abstract

The landscape of animal and plant breeding is rapidly evolving. Companies now invest millions in collecting extensive data on health, disease, welfare, and fertility, which are key fitness traits relevant for sustainable production. Likewise, genomic-based predictions or GWAS-based searches for causal variants in categorically scored psychiatric disorders and other diseases are key areas in clinical genetics. The categorical nature (yes/no, scores, etc.) of these traits violates the normality assumptions of standard linear models; threshold or threshold-linear models represent conceptually attractive alternatives. In Bayesian settings, these models can be fitted either via Gibbs sampling or by maximizing the posterior density (maximum a posteriori, MAP). While Gibbs sampling is flexible and can handle multivariate models with several categorical and continuous traits, its computational and memory demands limit its applicability in large-scale applications and genetic and genomic predictions. MAP methods are computationally efficient and suitable for large-scale datasets. However, existing MAP methods are restricted to a single categorical trait and many continuous ones, making them insufficient for the new generation of data. The lack of proper methods for fitting highly dimensional threshold-linear models for quantitative genetics has persisted for over 2 decades. Failing to model categorical traits appropriately can lead to reduced genetic gain and deterioration of the traits over time. This study develops theoretically sound and computationally efficient framework for jointly analyzing multiple categorical and Gaussian traits, specifically multi-trait threshold-linear models, including or not pedigree and genomic information. The proposed MAP multiple-trait threshold-linear model methods are based on Newton-Raphson and Expectation-Maximization schemes. Using a simulated dataset and treating Gibbs sampling as the benchmark, we found that the breeding values estimated by our methods agree nearly perfectly with those obtained from Gibbs sampling, while greatly reducing computational cost and allowing scalability to very large datasets. As expected, the Newton-Raphson iteration outperformed the Expectation-Maximization algorithm in terms of computational time. Our results demonstrate that routine genetic evaluations incorporating multiple categorical traits are now feasible using the presented methodology. Solving this decades-old gap enables accurate, large-scale genetic and genomic predictions of key categorical fitness traits under multi-trait models, unlocking the full potential of genomic selection for complex traits in animal and plant populations and genomic-based predictions in clinical genetics.

## Teaser

This study addresses a long-standing problem in genetics: how to analyze multiple categorical traits, such as disease status or fertility scores, together with continuous traits at large scale. Bayesian MCMC methods are either computationally unfeasible for large-scale applications or limited to a single categorical trait. Bermann et al. developed an efficient statistical framework to jointly analyze multiple categorical and continuous traits using threshold-linear models. Using simulations, the methods produced results nearly identical to benchmark approaches but required far less computation. The work enables routine, accurate genetic evaluations of key ordered categorical traits in agricultural breeding and clinical genetics.

## Introduction

Categorical traits such as disease resistance, resilience (eg survival, productive life, lodging), fertility (eg calving ease, litter size), health (eg disease resistance), welfare (eg skin damage), and other fitness-related traits are of interest in animal and plant breeding (Montesinos-López et al. 2014; Knap and Doeschl-Wilson 2020; Leite et al. 2023; Maskal et al. 2024). Likewise, genomic-based predictions or GWAS-based searches for causal variants in categorical psychiatric disorders and other diseases are key areas in clinical genetics (de los Campos et al. 2010; Visscher and Wray 2015; Mahjani et al. 2020; Albiñana et al. 2023; Lee et al. 2025). Genomic prediction for categorical traits is experiencing renewed interest, driven by the need for complex statistical models that can accommodate their discrete nature. In animal and plant breeding, selection on these traits is gaining momentum, to reduce production costs and environmental impact. Such traits may be binary, with 2 possible outcomes, or ordered categorical, with several ordered outcomes (Dempster and Lerner 1950; Gianola 1982; Falconer and Mackay 1996). In humans, complex statistical models with multiple traits and correlated random effects have been used to estimate genetic parameters and validate genomic predictions of psychiatry disorders (Mahjani et al. 2020; Albiñana et al. 2023).

Wright (1934) postulated the threshold model to consider traits of discrete distribution (number of fingers in Guinea pigs) that were nevertheless not due to single Mendelian determinism. This form of modeling allows inclusion of arbitrary fixed and random effects and can be extended to accommodate correlated continuous traits. In these models, the observed phenotype is assumed to result from discretizing an underlying, normally distributed liability through a set of thresholds (Wright 1934; Gianola and Foulley 1983). The number of thresholds is contingent upon the number of categories, with binary traits requiring a single threshold and traits with *m* categories requiring *m-1* thresholds. Threshold models are perceived as reflecting the distribution of categorical responses (Gianola 1982) while at the same time are flexible to integrate diverse information in a valid manner (Wang et al. 1997), eg covariance across relatives applies on the underlying scale, and models can include several effects. They can also be seen as a generalized linear model with a probit function. The outcome of genomic prediction using threshold models is an estimated breeding value (EBV) on the underlying scale that can be converted into a probability value (Gianola and Foulley 1983).

Genetic evaluation of threshold models is complicated because the estimators have nonlinear forms. They can be fitted in 2 main ways: by Bayesian Monte Carlo Markov Chain (MCMC) methods via the Gibbs sampler (Sorensen et al. 1995; Wang et al. 1997), or by maximum a posteriori (MAP) or posterior mode estimation (Gianola and Foulley 1983; Harville and Mee 1984). The Gibbs sampler draws samples from the posterior distribution of all parameters and unknowns in the model (variance components, thresholds, effects on the underlying scale) and is conceptually straightforward and simple to implement. However, its lengthy computing time, the memory demands for genomic predictions, and the need to evaluate whether the Markov chain has converged preclude its use in large-scale applications as typical in animal breeding.

MAP approaches using iterative solvers accommodate well much larger masses of data than MCMC and their convergence can easily be assessed. They estimate location parameters typically via Fisher-Scoring, where each iteration requires a series of rounds updating and solving a system of linear equations. Variance components are assumed known, usually from subsets where MCMC methods are feasible. Estimation of variance components can be performed with MAP approaches; however, MCMC methods are more robust, easy to apply and to generalize to different models. Although MAP approaches are suitable for large-scale applications (Foulley et al. 1983; Janss and Foulley 1993; Hoeschele et al. 1995), their main limitation is that theory developed so far can only accommodate a single categorical trait and cannot jointly model multiple categorical outcomes. For instance, a typical case is recording of the status (affected/not affected) of several diseases in the field at a given point in time. Consequently, genomic prediction for categorical traits cannot exploit genetic correlations among them, resulting in a lack of generality and suboptimal use of information.

Therefore, the objective of this study was to develop the first theoretically sound and computationally efficient framework for jointly analyzing multiple categorical and linear traits, specifically multi-trait threshold-linear models, with the possibility of including pedigree and genomic information (Aguilar et al. 2010). The proposed methods are based on Newton-Raphson and Expectation-Maximization schemes (ie all MAP iterative methods). These methods were validated by comparison with MCMC results.

## Theory

We propose 2 iterative schemes for the MAP estimator: Newton-Raphson and Expectation-Maximization. This section is divided into 3 subsections: 1 corresponding to the Newton-Raphson (NR) formulation, another one to the Expectation-Maximization (EM) algorithm, and the last 1 corresponding to computational details. For the sake of completeness and clarity, first derivations for models with a single categorical and multiple Gaussian traits are given, then we expand for the general case of several categorical traits. Henceforth, thresholds and variance components are assumed to be known. In the following, subscript 1 denotes categorical traits, whereas subscript 2 denotes continuous traits.

### Newton-Raphson algorithm

First, start assuming a single categorical trait and multiple linear traits. Here we recapitulate (until Eq. 16) known developments, mainly from Gianola and Foulley (1983), Quaas (1994), and Wang et al. (1997). The key results of this section lie in Eqs. (20, 23, 25, and 26).

The model is:


(1) $$\left[\begin{array}{c} l\\ y_{2} \end{array}\right]=\left[\begin{array}{cc} W_{1} & 0\\ 0 & W_{2} \end{array}\right]\left[\begin{array}{c} \theta_{1}\\ \theta_{2} \end{array}\right]+\left[\begin{array}{c} e_{1}\\ e_{2} \end{array}\right]$$

where l is the vector of the unobserved liability of the categorical trait $\left(y_{1}\right)$, $y_{2}$ is the vector containing phenotypes for continuously distributed traits, $W_{i}=\left[\begin{array}{cc} X_{i} & Z_{i} \end{array}\right]$ is an incidence matrix, and ${\theta^{\prime }}_{i}=\left[\begin{array}{cc} {b^{\prime }}_{i} & {u^{\prime }}_{i} \end{array}\right]$, where $b^{\prime }=\left[\begin{array}{cc} {b^{\prime }}_{1} & {b^{\prime }}_{2} \end{array}\right]$ is the vector of fixed effects and $u=\left[\begin{array}{cc} {u^{\prime }}_{1} & {u^{\prime }}_{2} \end{array}\right]$ is the vector of random effects distributed as u∼N(0,G); e is the vector of errors, distributed as e∼N(0,R⊗I), where $R=\left[\begin{array}{cc} r_{11} & r_{12}\\ r_{21} & R_{22} \end{array}\right]$.

The log posterior density for the model (1) is:


(2) $$\log\;L(\theta |y_{1},y_{2})=\log\;L(y_{1}|y_{2},\;\theta )+\log\;L(y_{2}|\theta )+\log\;L(\theta )+const$$

The estimate of θ can be obtained by maximizing (2), being a joint posterior mode (or MAP) from a Bayesian perspective. Note that this way of estimating θ follows the same logic as C.R. Henderson when deriving BLUP (Henderson, 1950).

One way to maximize $\log\;L(\theta |y_{1},y_{2})$ is by the Newton-Raphson algorithm, whose iterative scheme is:


(3) $$\theta^{\;j+1}=\theta^{j}-H{\left(j\right)}^{-1}\nabla (j)$$

where H(j) and ∇(j) are the Hessian and gradient of $\log\;L(\theta |y_{1},y_{2})$ evaluated at iteration *j*, respectively.

To apply Eq. (3), we start by noting that since the elements of $y_{1}$ in the right-hand side of Eq. (2) are assumed to be conditionally independent given θ, $\log\;L(y_{1}|y_{2},\;\theta )$ can be written as the sum of the log-likelihoods of each $y_{1_{i}}$. Such distribution is multinomial with a single trial, with probabilities of each of the *K* possible events described by the underlying liability (*l*) falling in between 2 thresholds ($t_{k}$ and $t_{k-1}$). This can be written as:


(4) $$p(y_{1_{i}}|y_{2_{i}},\;\theta )=\overset{K}{\underset{k=1}{\prod }}\;P_{ki}1_{y_{1_{i}}}=k=\overset{K}{\underset{k=1}{\prod }}\;(\Phi (\eta_{k_{i}})-\Phi (\eta_{k-1_{i}}){)}^{1_{y_{1_{i}}=k}}$$

where Φ(⋅) is the standard normal cumulative distribution function and $\eta_{k_{i}}=\frac{t_{k}-\mu_{i}}{\sigma_{i}}$, $t_{k}$ being the upper threshold for the *k^th^* category, $\mu_{i}=E[l_{i}|y_{2_{i}},\;\theta ]$ the conditional expected value of the liability, and $\sigma_{i}^{2}=\mathrm{Var}(l_{i}|y_{2_{i}},\;\theta )$ its variance. The operator $1_{y_{1_{i}}=k}$ equals 1 when $y_{1_{i}}=k$ (the phenotype is the *k*-th category) and 0 otherwise. By properties of multivariate normality, $\mu_{i}=W_{1_{i}}\theta_{1}+r_{12}R_{22}^{-1}(y_{2_{i}}-W_{2_{i}}\theta_{2})$ and $\sigma_{i}^{2}=r_{11}-r_{12}R_{22}^{-1}r_{21}$.

Then, Eq. (2) becomes:


(5) $$\begin{array}{l} \log L\left(\theta |y_{1},y_{2}\right)=\sum_{i=1}^{n}\sum_{k=1}^{K}1_{y_{1_{j}}}=k\log\left(P_{k_{i}}\right)+\mathrm{const}\\ \;-\frac{1}{2}{\left(y_{2}-W_{2}\theta \right)}^{\prime }{\left(R_{22}⊗I\right)}^{-1}\left(y_{2}-W_{2}\theta \right)+\mathrm{const}-\frac{1}{2}\theta^{\prime }\mathrm{S\theta}+const \end{array}$$

where $S=\left[\begin{array}{cc} 0 & 0\\ 0 & G^{-1} \end{array}\right]$.

The first partial derivative of each $\log\;(P_{k_{i}})$ is (Quaas 1994):


(6) $$\frac{\partial }{\partial \theta^{\prime }}\log\;(P_{k_{i}})=\frac{1}{P_{k_{i}}\sigma_{i}}{{\tilde{W}}^{\prime }}_{i}(-\phi (\eta_{k_{i}})+\phi (\eta_{k-1_{i}}))={{\tilde{W}}^{\prime }}_{i}\delta_{i}$$

where ϕ(⋅) is the standard normal probability density function, $\delta_{i}=\frac{1}{\sigma_{i}}\frac{-\phi (\eta_{k_{i}})+\phi (\eta_{k-1_{i}})}{P_{k_{i}}}$ and ${{\tilde{W}}^{\prime }}_{i}={\left[\begin{array}{cc} W_{1_{i}} & -r_{12}R_{22}^{-1} \end{array}W_{2_{i}}\right]}^{\prime }$ has the appropriate dimensions to be conformable with the other likelihood terms. Then, the first partial derivative of $\log\;L(\theta |y_{1},y_{2})$ with respect to θ is:


(7) $$\begin{array}{rl} \frac{\partial }{\partial \theta^{\prime }}\log\;L(\theta |y_{1},y_{2})= & \overset{n}{\underset{i=1}{\sum }}\;\overset{K}{\underset{k=1}{\sum }}\;1_{y_{1_{i}}=k}{{\tilde{W}}^{\prime }}_{i}\delta_{i}\\ & +{W^{\prime }}_{2}(R_{22}⊗I{)}^{-1}(y_{2}-W_{2}\theta )-S\theta \end{array}$$

Taking the second derivative of each element of the double summation gives (Quaas 1994):


(8) $$\frac{\partial }{\partial \theta }{{\tilde{W}}^{\prime }}_{i}\delta_{i}={{\tilde{W}}^{\prime }}_{i}\left[\frac{-\phi (\eta_{k_{i}})}{P_{k_{i}}\sigma_{i}^{2}}\eta_{k_{i}}+\frac{\phi (\eta_{k-1_{i}})}{P_{k_{i}}\sigma_{i}^{2}}\eta_{k-1_{i}}-{\left(\frac{-\phi (\eta_{k_{i}})+\phi (\eta_{k-1_{i}})}{P_{k_{i}}\sigma_{i}}\right)}^{2}\right]{\tilde{W}}_{i}=-\gamma_{i}{{\tilde{W}}^{\prime }}_{i}{\tilde{W}}_{i}$$

where $\gamma_{i}$ is the negative of the expression inside the square brackets. Note that the final minus sign is for convenience. Finally, the second partial derivative of $\log\;L(\theta |y_{1},y_{2})$ with respect to θ is:


(9) $$\begin{array}{ll} \frac{\partial^{2}}{\partial \text{θ}\partial {\text{θ}}^{\prime }}\log L\left(\text{θ}|{\text{y}}_{1},{\text{y}}_{2}\right)= & \sum_{i=1}^{n}\sum_{k=1}^{K}1_{{\text{y}}_{1_{i}}}=k\left(-\gamma_{i}{\tilde{W}}^{\prime }{\tilde{W}}_{i}\right)\\ & -{{\text{W}}^{\prime }}_{2}{\left({\text{R}}_{22}⊗\text{I}\right)}^{-1}{\text{W}}_{2}-\text{S} \end{array}$$

Now, the contribution of the *i*th animal with records to the gradient $\nabla =\frac{\partial }{\partial \theta^{\prime }}\log\;L(\theta |y_{1},y_{2})$ is:


(10) $$\nabla_{i}=\left[\begin{array}{cc} {W^{\prime }}_{1_{i}} & 0\\ 0 & {W^{\prime }}_{2_{i}} \end{array}\right]\left[\begin{array}{c} \delta_{i}\\ -R_{22}^{-1}r_{21}\delta_{i}+R_{22}^{-1}(y_{2_{i}}-W_{2_{i}}\theta_{i}) \end{array}\right]={W^{\prime }}_{i}z_{i}$$

and for the Hessian $H=\frac{\partial^{2}}{\partial \theta \partial \theta^{\prime }}\log\;L(\theta |y_{1},y_{2})\;$ one gets:


(11) $$\begin{array}{rl} H_{i} & =-\gamma_{i}{{\tilde{W}}^{\prime }}_{i}{\tilde{W}}_{i}-{W^{\prime }}_{2}R_{22}^{-1}W_{2}\\ & =-\left[\begin{array}{cc} {W^{\prime }}_{1i} & 0\\ 0 & {W^{\prime }}_{2i} \end{array}\right]\left[\begin{array}{cc} \gamma_{i} & -\gamma_{i}r_{12}R_{22}^{-1}\\ -\gamma_{i}R_{22}^{-1}r_{21} & \gamma_{i}R_{22}^{-1}r_{21}r_{12}R_{22}^{-1}+R_{22}^{-1} \end{array}\right]\left[\begin{array}{cc} W_{1i} & 0\\ 0 & W_{2i} \end{array}\right]\\ & =-{W^{\prime }}_{i}{\tilde{R}}_{i}^{-1}W_{i} \end{array}$$

where ${\tilde{R}}_{i}^{-1}=\left[\begin{array}{cc} \gamma_{i} & -\gamma_{i}r_{12}\;R_{22}^{-1}\\ -\gamma_{i}R_{22}^{-1}r_{21} & \gamma_{i}R_{22}^{-1}r_{21}r_{12}R_{22}^{-1}+R_{22}^{-1} \end{array}\right]$. Then, from Eq. (10) and Eq. (11) the full gradient and Hessian at iteration *j* are:


(12) $$\nabla (j)=\overset{n}{\underset{i=1}{\sum }}\;\nabla_{i}(j)-S\theta^{j}=\overset{n}{\underset{i=1}{\sum }}\;{W^{\prime }}_{i}z_{i}^{j}-S\theta^{j}=W^{\prime }z^{j}-S\theta^{j}$$

and


(13) $$H(j)=\overset{n}{\underset{i=1}{\sum }}-{W^{\prime }}_{i}{\tilde{R}}_{i}^{-1}W_{i}-S=-(W^{\prime }{\tilde{R}}^{-1}W+S)$$

Plugging Eqs. (12) and (13) into Eq. (3) and by the reasoning of Quaas (1994; p. 163–164) gives the following system of equations:


(14) $$\left(W^{\prime }{\tilde{R}}^{-1}W+S)\theta^{\;j+1}=W^{\prime }{\tilde{R}}^{-1}(W\theta^{j}+\tilde{R}z^{j}\right)$$

which resembles the linear mixed model equations with $\tilde{R}$ as residual covariance matrix and $\left(W\theta^{j}+\tilde{R}z^{j}\right)$ as pseudo-observations. By noting that ${\tilde{R}}_{i}=\left[\begin{array}{cc} \gamma_{i}^{-1}+r_{12}R_{22}^{-1}r_{21} & r_{12}\\ r_{21} & R_{22} \end{array}\right]$, we have:


(15) $$W\theta +{\tilde{R}}_{i}z_{i}=\left[\begin{array}{c} \gamma_{i}^{-1}\delta_{i}+W_{1_{i}}\theta_{i}+r_{12}R_{22}^{-1}(y_{2_{i}}-W_{2_{i}}\theta_{i})\\ y_{2_{i}} \end{array}\right]=\left[\begin{array}{c} {\hat{y}}_{1_{i}}\\ y_{2_{i}} \end{array}\right]={\tilde{y}}_{i}$$

which defines $\tilde{y}$. Finally, the system of equations in Eq. (14) is:


(16) $$(W^{\prime }{\tilde{R}}^{-1}W+S)\theta^{\;j+1}=W^{\prime }{\tilde{R}}^{-1}\tilde{y}$$

The iterative scheme works by at each round: (i) for each animal with records, calculate $\delta_{i}$, $\gamma_{i}$, ${\tilde{R}}_{i}^{-1}$, and ${\tilde{y}}_{i}$ from Eq. (6, 8, 11, and 15), respectively; (ii) solve mixed model equations from Eq. (16); and (iii), repeat until convergence. Readers are referred to Quaas (1994) for further details.

New developments start at this point. Now, we expand the model to an arbitrary number of categorical traits (say, *c*). Let l in Eq. (1) be the vector of the unobserved liabilities for these traits. The remaining matrices and vectors of the model keep their definition except from R, which changes to $R=\left[\begin{array}{cc} R_{11} & R_{12}\\ R_{21} & R_{22} \end{array}\right]$. The log-likelihood of Eq. (2) has the same form and the records of different individuals are treated as conditionally independent in $\log\;L(y_{1}|y_{2},\;\theta )$. However, the traits within an individual with records are not independent; therefore, their joint probability must be considered in Eq. (2).

Let the scores for categorical traits for the *i*th animal be comprised in $y_{1_{i}}$, where each $y_{1_{ij}}$ is the observation corresponding to the realization of $t_{\;j_{k-1}}<l_{ij}\leq t_{\;j_{k}}$, with $t_{\;j_{k}}$ being the upper threshold for the *j*th trait and *k*th category. Then, the distribution of $y_{1_{i}}$ conditional on the continuous phenotypes and solutions is:


(17) $$\begin{array}{rl} p(y_{1_{i}}|y_{2_{i}},\;\theta ) & =\underset{k\in K}{\prod }\;P_{i}^{1_{y_{1_{i}}=\;k}}=\underset{k\in K}{\prod }\;P(t_{k-1}<l_{i}\leq t_{k}{)}^{1_{y_{i_{i}}=\;k}}\\ & =\underset{k\in K}{\prod }\;{\left(\int_{T_{k}}\phi (l_{i};\mu_{i},\Sigma_{i})dl_{i}\right)}^{1_{y_{1_{i}}=\;k}} \end{array}$$

where k is a vector containing a combination of the different categorical traits, K is the set of all possible k‘s, $T_{k}$ is the hyper-rectangle determined by the vector of upper $\left(t_{k}\right)$ and lower $\left(t_{k-1}\right)$ thresholds of each k, and $\phi (l_{i};\mu_{i},\Sigma_{i})$ is the multivariate normal density with mean $\mu_{i}=E[l_{i}|y_{2_{i}},\;\theta ]$ and variance $\Sigma_{i}=\mathrm{Var}(l_{i}|y_{2_{i}},\;\theta )$ evaluated at $l_{i}$. In this case, again by multivariate normality, $\mu_{i}=W_{1_{i}}\theta_{1}+R_{12}R_{22}^{-1}(y_{2_{i}}-W_{2_{i}}\theta_{2})$ and $\Sigma_{i}=R_{11}-R_{12}R_{22}^{-1}R_{21}$.

Then, the log joint posterior from Eq. (2) is:


(18) $$\log\;L(\theta |y_{1},y_{2})=\overset{n}{\underset{i=1}{\sum }}\;\underset{k\in K}{\sum }\;1_{y_{1_{i}}=\;k}\log\;P_{i}+const-\frac{1}{2}(y_{2}-W_{2}\theta {)}^{\prime }(R_{22}⊗I{)}^{-1}(y_{2}-W_{2}\theta )+const-\frac{1}{2}\theta^{\prime }S\theta +const$$

To get the first partial derivative of each $\log\;P_{i}$ with respect to θ, first differentiate Eq. (17) under the integral sign:


(19) $$\frac{\partial }{\partial \theta^{\prime }}\log\;(P_{i})=\frac{1}{P_{i}}\int_{T_{k}}\frac{\partial }{\partial \theta^{\prime }}\phi (l_{i};\mu_{i},\Sigma_{i})dl_{i}$$

After algebra (see Appendix 1):


(20) $$\begin{array}{rl} \frac{\partial }{\partial \theta^{\prime }}\log\;(P_{i}) & ={{\tilde{W}}^{\prime }}_{i}\Sigma_{i}^{-1}\int_{T_{k}}(l_{i}-\mu_{i})\phi_{T}(l_{i};\mu_{i},\Sigma_{i})dl_{i}\\ & =\;{{\tilde{W}}^{\prime }}_{i}\Sigma_{i}^{-1}(E_{T}[l_{i}]-\mu_{i})={{\tilde{W}}^{\prime }}_{i}\Delta_{i} \end{array}$$

where, as before, ${{\tilde{W}}^{\prime }}_{i}={\left[\begin{array}{c} W_{1_{i}}-R_{12}R_{22}^{-1} \end{array}W_{2_{i}}\right]}^{\prime }$, and $E_{T}[l_{i}]$ denotes the expectation of $l_{i}$ distributed as a truncated multivariate normal vector with mean $\mu_{i}$, variance $\Sigma_{i}$, and truncation region $T_{k}$. Thus, $\Delta_{i}=\Sigma_{i}^{-1}(E_{T}[l_{i}]-\mu_{i})$ is the multivariate analog of $\delta_{i}$ in Eq. (6). Note that $E_{T}[l_{i}]$ has no closed-form expression.

Then, the first derivative of the log joint posterior in (19) with respect to θ is:


(21) $$\begin{array}{rl} \frac{\partial }{\partial \theta^{\prime }}\log\;L(\theta |y_{1},y_{2}) & =\overset{n}{\underset{i=1}{\sum }}\;\underset{k\in K}{\sum }\;1_{y_{1_{i}}=\;k}{W^{\prime }}_{i}\Delta_{i}\\ & +{W^{\prime }}_{2}(R_{22}⊗I{)}^{-1}(y_{2}-W_{2}\theta )-S\theta \end{array}$$

To calculate the second derivative of Eq. (18), start from Eq. (20) and re-arrange the expectation:


(22) $$\frac{\partial }{\partial \theta }{{\tilde{W}}^{\prime }}_{i}\Delta_{i}={{\tilde{W}}^{\prime }}_{i}\frac{\partial }{\partial \theta }E_{T}[\Sigma_{i}^{-1}(l_{i}-\mu_{i})]={{\tilde{W}}^{\prime }}_{i}\int_{T_{k}}\frac{\partial }{\partial \theta }\Sigma_{i}^{-1}(l_{i}-\mu_{i})\phi_{T}(l_{i};\mu_{i},\Sigma_{i})dl_{i}$$

Applying the product rule inside the integrand as shown in the Appendix 2:


(23) $$\frac{\partial }{\partial \theta }{{\tilde{W}}^{\prime }}_{i}\Delta_{i}=-{{\tilde{W}}^{\prime }}_{i}(\Sigma_{i}^{-1}-\Sigma_{i}^{-1}\mathrm{Va}r_{T}(l_{i})\Sigma_{i}^{-1}){\tilde{W}}_{i}=-{{\tilde{W}}^{\prime }}_{i}\Gamma_{i}{\tilde{W}}_{i}$$

where $\mathrm{Va}r_{T}(l_{i})$ denotes the covariance matrix of $l_{i}$ distributed as a truncated multivariate normal vector with mean $\mu_{i}$, variance $\Sigma_{i}$, and truncation region $T_{k}$. Note that $\mathrm{Va}r_{T}(l_{i})$ has no closed-form expression but can be computed numerically. Then, $\Gamma_{i}=\Sigma_{i}^{-1}-\Sigma_{i}^{-1}\mathrm{Va}r_{T}(l_{i})\Sigma_{i}^{-1}$ is the multivariate analog of $\gamma_{i}$ in Eq. (8). Then, the second partial derivative of the log joint posterior is:


(24) $$\begin{array}{cc} \frac{\partial }{\partial {\text{θ}}^{\prime }\partial \text{θ}}\log L\left(\text{θ}|{\text{y}}_{1},{\text{y}}_{2}\right)= & \sum_{i=1}^{n}\sum_{k\in K}1_{{\text{y}}_{1_{i}}}=k\left(-{{\tilde{\text{W}}}^{\prime }}_{i}\Gamma_{i}{\tilde{\text{W}}}_{i}\right)\\ & +{{\text{W}}^{\prime }}_{2}{\left({\text{R}}_{22}⊗\text{I}\right)}^{-1}{\text{W}}_{2}-\text{S} \end{array}$$

The contribution of the *i^th^* animal with records to the gradient and Hessian gives:


(25) $$\nabla_{i}=\left[\begin{array}{cc} {W^{\prime }}_{1_{i}} & 0\\ 0 & {W^{\prime }}_{2_{i}} \end{array}\right]\left[\begin{array}{c} \Delta_{i}\\ -R_{22}^{-1}R_{21}\Delta_{i}+R_{22}^{-1}(y_{2_{i}}-W_{2_{i}}\theta_{i}) \end{array}\right]={W^{\prime }}_{i}z_{i}$$

and:


(26) $$H_{i}=-{{\tilde{W}}^{\prime }}_{i}\Gamma_{i}{\tilde{W}}_{i}-{W^{\prime }}_{2}R_{22}^{-1}W_{2}=-\left[\begin{array}{cc} {W^{\prime }}_{1i} & 0\\ 0 & {W^{\prime }}_{2i} \end{array}\right]\left[\begin{array}{cc} \Gamma_{i} & -\Gamma_{i}R_{12}R_{22}^{-1}\\ -R_{22}^{-1}R_{21}\Gamma_{i} & R_{22}^{-1}R_{21}\Gamma_{i}R_{12}R_{22}^{-1}+R_{22}^{-1} \end{array}\right]\left[\begin{array}{cc} W_{1i} & 0\\ 0 & W_{2i} \end{array}\right]=-{W^{\prime }}_{i}{\tilde{R}}_{i}^{-1}W_{i}$$

The full gradient and Hessian are as in Eq. (12) and (13), respectively. After algebra, the iteration in Eq. (3) gives the same mixed model equations as in Eq. (14). In this case, with ${\tilde{R}}_{i}=\left[\begin{array}{cc} \Gamma_{i}^{-1}+R_{12}R_{22}^{-1}R_{21} & R_{12}\\ R_{21} & R_{22} \end{array}\right]$, gives:


(27) $$\begin{array}{rl} W\theta +{\tilde{R}}_{i}z_{i} & =\left[\begin{array}{c} \Gamma_{i}^{-1}\Delta_{i}+W_{1_{i}}\theta_{i}+R_{12}R_{22}^{-1}(y_{2_{i}}-W_{2_{i}}\theta_{i})\\ y_{2_{i}} \end{array}\right]\\ & =\left[\begin{array}{c} {\hat{y}}_{1_{i}}\\ y_{2_{i}} \end{array}\right]={\tilde{y}}_{i} \end{array}$$

as pseudo-data vector. The mixed model equations are $(W^{\prime }{\tilde{R}}^{-1}W+S)\theta^{\;j+1}=W^{\prime }{\tilde{R}}^{-1}\tilde{y}$, and the iterative scheme works as follows: (i) for each animal with records, calculate $\Delta_{i}$, $\Gamma_{i}$, ${\tilde{R}}_{i}^{-1}$, and ${\tilde{y}}_{i}$ from Eq. (20, 23, 26, and 27), respectively; (ii) solve mixed model equations from Eq. (16); and (iii), repeat until convergence.

### Expectation-maximization algorithm

As for the Newton-Raphson scheme, we start by specifying the model for a single categorical trait and an arbitrary number of continuous traits. In this section, there is no novelty until after Eq. (36). The most important results of this section are in Eqs. (39, 40).

The model specification is the same as in Eq. (1). For the Expectation-Maximization algorithm, 1 needs to define the complete and observed data (Dempster et al. 1977). The complete data should be defined such that inference based on it is simpler than based on the observed data. After defining the complete data, its likelihood should be formulated. Finally, the algorithm works by iterating until convergence between the E-step (taking the expectation of the complete likelihood conditional on the observed data) and the M-step (maximizing the likelihood obtained in the E-step and updating the vector of unknowns of interest).

For the threshold-linear model, the complete and observed data are $\left(l,y_{2}\right)$ and $\left(y_{1},y_{2}\right)$, respectively. The complete penalized log likelihood is:


(28) $$\log\;L(\theta |l,y_{2})=\log\;L(l|y_{2},\;\theta )+\log\;L(y_{2}|\theta )+\log\;L(\theta )$$

Note that Eq. (28) only involves multivariate normal distributions, as an ordinary linear mixed model. The second and third term of the right-hand side of Eq. (28) do not involve the observed data, $y_{1}$. Thus, the E-step at iteration *j* is:


(29) $$\begin{array}{rl} Q(\theta |\theta^{j}) & =E_{l|y_{1},else}[\log\;L(\theta |l,y_{2})]=E_{l|y_{1},else}[\log\;L(l|y_{2},\;\theta )]\\ & +\log\;L(y_{2}|\theta )+\log\;L(\theta ) \end{array}$$

where $Q(\theta |\theta^{j})$ is the expected value of $\log\;L(\theta |l,y_{2})$ given the observed data and parameters estimated in the previous iteration. Thus:


(30) $$\begin{array}{r} E_{l|y_{1},else}[\log\;L(l|y_{2},\;\theta )]=E_{l|y_{1},else}\left[\overset{n}{\underset{i=1}{\sum }}\;\log\;L(l_{i}|y_{2},\;\theta )\right]\\ =\overset{n}{\underset{i=1}{\sum }}\;E_{l|y_{1},else}[\log\;L(l_{i}|y_{2},\;\theta )] \end{array}$$

As in Eq. (4), the log-likelihood is normal with mean $\mu_{i}$ and variance $\sigma_{i}^{2}$. Then, the *i^th^* term of the right-hand side of the Eq. (30) is:


(31) $$\begin{array}{rl} E_{l|y_{1},else}[\log\;L(l_{i}|y_{2},\;\theta )] & =-\frac{1}{2}\log\;(2\pi \sigma_{i}^{2})-\frac{1}{2\sigma_{i}^{2}}({\tilde{l}}_{i}-\mu_{i}{)}^{2}\\ & -\frac{1}{2\sigma_{i}^{2}}\mathrm{Va}r_{l|y_{1},else}(l_{i}) \end{array}$$

where:


(32) $${\tilde{l}}_{i}=\mu_{i}-\sigma_{i}\frac{\phi (\eta_{k_{i}})-\phi (\eta_{k-1_{i}})}{\Phi (\eta_{k_{i}})-\Phi (\eta_{k-1_{i}})}$$

Note that the first 2 terms of the right-hand side of Eq. (31) look like a normal likelihood with ${\tilde{l}}_{i}$ as observation, $\mu_{i}$ as mean, and $\sigma_{i}^{2}$ as variance. Therefore:


(33) $$E_{l|y_{1},else}[\log\;L(l_{i}|y_{2},\;\theta )]=\log\;L({\tilde{l}}_{i}|y_{2},\;\theta )-\frac{1}{2\sigma_{i}^{2}}\mathrm{Va}r_{l|y_{1},else}(l_{i})$$

Then, coming back to Eq. (30):


(34) $$\begin{array}{rl} E_{l|y_{1},else}[\log\;L(l|y_{2},\;\theta )]= & \overset{n}{\underset{i=1}{\sum }}\;\log\;L({\tilde{l}}_{i}|y_{2},\;\theta )-\frac{1}{2\sigma_{i}^{2}}\mathrm{Va}r_{l|y_{1},else}(l_{i})\\ & =\log\;L(\tilde{l}|y_{2},\;\theta )-\frac{1}{2}\overset{n}{\underset{i=1}{\sum }}\frac{1}{\sigma_{i}^{2}}\mathrm{Va}r_{l|y_{1},else}(l_{i}) \end{array}$$

Plugging this result into Eq. (29):


(35) $$Q(\theta |\theta^{j})=E_{l|y_{1},else}[\log\;L(\theta |l,y_{2})]=\log\;L(\tilde{l}|y_{2},\;\theta )-\frac{1}{2}\overset{n}{\underset{i=1}{\sum }}\frac{1}{\sigma_{i}^{2}}\mathrm{Va}r_{l|y_{1},else}(l_{i})+\log\;L(y_{2}|\theta )+\log\;L(\theta )$$

For the M-step, only the terms in Eq. (35) involving θ need to be considered. Note that in $\log\;L(\tilde{l}|y_{2},\;\theta )$ only the $\mu_{i}$ ’s depend on θ because the ${\tilde{l}}_{i}$ ’s depend on $\theta^{j}$ (the current solution) but not on θ (the true, exact solution). By a similar argument, $\frac{1}{2}\overset{n}{\underset{i=1}{\sum }}\;\frac{1}{\sigma_{i}^{2}}\mathrm{Va}r_{l|y_{1},else}(l_{i})$ is treated as a constant for the M-step and can be ignored. Then, the M-step is:


(36) $$\begin{array}{rl} \mathrm{argma}x_{\theta }\;Q(\theta |\theta^{j})\;= & \;\mathrm{argma}x_{\theta }\log\;L(\tilde{l}|y_{2},\;\theta )+\log\;L(y_{2}|\theta )\\ & +\log\;L(\theta ) \end{array}$$

which can be done by solving a regular linear mixed model using $\left(\tilde{l},y_{2}\right)$ as observations. Thus, the iterative scheme for the EM threshold-linear model is: (i) for each animal with records, calculate ${\tilde{l}}_{i}$ as in Eq. (32) (E-step); (ii) solve mixed-model equations using $\left(\tilde{l},y_{2}\right)$ as phenotypes (M-step); and (iii), repeat until convergence. This was used, for instance, by Wang et al. (1997).

The novelty of this work is extending the EM threshold-linear model to multiple categorical traits. This is simpler than for the Newton-Raphson formulation. Eq. (29) still holds, but Eq. (30) is modified to consider all the traits for the *i*th animal with observations at the same time:


(37) $$E_{l|y_{1},else}[\log\;L(l|y_{2},\;\theta )]=\overset{n}{\underset{i=1}{\sum }}\;E_{l|y_{1},else}[\log\;L(l_{i}|y_{2},\;\theta )]$$

Here, the log-likelihood is multivariate normal with mean $\mu_{i}$ and variance $\Sigma_{i}$. Then:


(38) $$E_{l|y_{1},else}[\log\;L(l_{i}|y_{2},\;\theta )]=E_{l|y_{1},else}\left[-\frac{c}{2}\log\;(2\pi )-\frac{1}{2}\log\;|\Sigma_{i}|-\frac{1}{2}{\left(l_{i}-\mu_{i}\right)}^{\prime }\Sigma_{i}^{-1}(l_{i}-\mu_{i})\right]$$

Adding and subtracting $E_{l|y_{1},else}[{l^{\prime }}_{i}]\Sigma_{i}^{-1}E_{l|y_{1},else}[l_{i}]$ gives:


(39) $$\begin{array}{rl} E_{l|y_{1},else}[\log\;L(l_{i}|y_{2},\;\theta )]= & -\frac{c}{2}\log\;(2\pi )-\frac{1}{2}\log\;|\Sigma_{i}|\\ & -\frac{1}{2}({\tilde{l}}_{i}-\mu_{i}{)}^{\prime }\Sigma_{i}^{-1}({\tilde{l}}_{i}-\mu_{i})-\frac{1}{2}C \end{array}$$

where $C=E_{l|y_{1},else}[{l^{\prime }}_{i}\Sigma_{i}^{-1}l_{i}]-E_{l|y_{1},else}[{l^{\prime }}_{i}]\Sigma_{i}^{-1}E_{l|y_{1},else}[l_{i}]$. Then, $E_{l|y_{1},else}[l_{i}]$ is:


(40) $$E_{l|y_{1},else}[l_{i}]={\tilde{l}}_{i}$$

Which can be understood as the expectation of the liability given the observed data and parameters of the model. Similar as Eq. (20), ${\tilde{l}}_{i}$ is a function of the truncated multivariate normal probability cumulative function, and it can be computed numerically. The first 3 terms of the right-hand side of Eq. (39) look like a multivariate normal likelihood. Thus:


(41) $$E_{l|y_{1},else}[\log\;L(l_{i}|y_{2},\;\theta )]=\log\;L({\tilde{l}}_{i}|y_{2},\;\theta )-\frac{1}{2}C$$

And Eq. (37) results in:


(42) $$\begin{array}{rl} E_{l|y_{1},else}[\log\;L(l|y_{2},\;\theta )] & =\overset{n}{\underset{i=1}{\sum }}\;\log\;L({\tilde{l}}_{i}|y_{2},\;\theta )-\frac{1}{2}C\;\\ & =\log\;L(\tilde{l}|y_{2},\;\theta )-\frac{1}{2}\overset{n}{\underset{i=1}{\sum }}\;C\; \end{array}$$

Finally, the E-step is:


(43) $$\begin{array}{rl} Q(\theta |\theta^{j}) & =E_{l|y_{1},else}[\log\;L(l,y_{2},\theta )]\\ & =\log\;L(\tilde{l}|y_{2},\;\theta )-\frac{1}{2}\overset{n}{\underset{i=1}{\sum }}\;C\;+\log\;L(y_{2}|\theta )+\log\;L(\theta ) \end{array}$$

By a similar argument as for Eq. (36), the M-step can be achieved by solving a linear mixed model using $\dot{y}=(\tilde{l},y_{2})$ as observations. Note that $\dot{y}\neq \tilde{y}$ from Eq. (27). Therefore, the iterative scheme for the EM threshold-linear model with multiple categorical traits is: (i) for each animal with records, calculate ${\tilde{l}}_{i}$ as in Eq. (40) (E-step); (ii) solve mixed-model equations $(W^{\prime }R^{-1}W+S)\theta^{\;j+1}=W^{\prime }R^{-1}\dot{y}$ using $\dot{y}$ as phenotypes (M-step); and (iii), repeat until convergence.

### Application details

For more than 1 categorical trait, $E_{T}[l_{i}]={\tilde{l}}_{i}$ (used both in EM and in NR) and $\mathrm{Va}r_{T}(l_{i})$ (used in NR) do not have a closed form. Therefore, they must be calculated numerically. Manjunath and Wilhelm (2021) derived the moments of the multivariate truncated normal distribution and provided an algorithm for calculating them. Their expression for the mean in the region determined by $t_{k-1}$ and $t_{k}$ is:


(44) $$E_{T}[l_{i}]=\mu_{i}+\Sigma_{i}(F_{i}(t_{k-1})-F_{i}(t_{k}))$$

where $F_{i}$ is a vector of marginal normal densities evaluated at its argument. Eq. (44) corresponds to formulas (11) and (17) of Manjunath and Wilhelm (2021). The formula for the variance is:


(45) $$\mathrm{Va}r_{T}(l_{i})=\Sigma_{i}+\Sigma_{i}P_{i}\Sigma_{i}+Q_{i}-E_{T}[l_{i}]E_{T}[{l^{\prime }}_{i}]$$

where $P_{i}$ is a diagonal matrix such that $P_{i_{qq}}=\frac{t_{k-1_{q}}F_{i_{q}}(t_{k-1})-t_{k_{q}}F_{i_{q}}(t_{k})}{\Sigma_{i_{qq}}}$, $t_{k_{q}}\;$ is the *q*th element of vector $t_{k}\;F_{i_{q}}$ is the *q*th row of matrix $F_{i}$ and $Q_{i}$ is a matrix such that $Q_{i_{\;jk}}=\overset{c}{\underset{l=1}{\sum }}\;\sigma_{\;jl}\underset{q\neq l}{\sum }\;\left(\sigma_{kq}-\frac{\sigma_{ql}\sigma_{lk}}{\sigma_{ll}}\right)M_{lq}\;$, where $\sigma_{ij}$ denote the *ij*th element of $\Sigma_{i}$ and $M_{lq}$ is a scalar of differences of bivariate marginal normal densities evaluated at the thresholds. Eq. (45) corresponds to formulas (16) and (18) of Manjunath and Wilhelm (2021). Calculating $F_{i}$ and $Q_{i}$ requires evaluating the integral of the multivariate normal distribution, for which the algorithm of Miwa et al. (2003) that uses a recursive integration method was chosen.

The EM algorithm only requires computing $E_{T}[l_{i}]$, whereas the NR algorithm requires both $E_{T}[l_{i}]$ and $\mathrm{Va}r_{T}(l_{i})$. The calculation of Eq. (44) is reasonably simple and computationally cheap. However, Eq. (45) is orders of magnitude slower than Eq. (44) because it requires significantly more evaluations of multivariate normal probabilities due to $Q_{i}$. Thus, although with a smaller number of iterations, a naïve implementation with iteration on data of the NR algorithm could be slower than the EM algorithm because of heavier computations when solving the mixed-model equations in Eq. (16). Noting that ${\tilde{R}}^{-1}$ does not change when solving Eq. (16) makes it possible to precompute it only once per Newton-Raphson iteration, hence, notably reducing the computational cost of Eq. (16) in iteration on data schemes. This is critical for an efficient Newton-Raphson implementation using iteration on data.

The convergence of the algorithms was assessed by $\frac{{\left\|e\right\|}^{2}}{{\left\|W^{\prime }{\tilde{R}}^{-1}\tilde{y}\right\|}^{2}}<10^{-12}$, where $e=W^{\prime }{\tilde{R}}^{-1}\tilde{y}-(W^{\prime }{\tilde{R}}^{-1}W+S)\theta^{j}$ (ie the residual of plugging the solutions of the previous round into Eq. (16)). The SQUAREM acceleration (Varadhan and Roland 2008) was applied every other round to the EM algorithm to improve convergence. All the methods presented in this study were implemented in Fortran 90/95 and included in blupf90+ and blup90iod3 from the Blupf90 software suite (Lourenco et al. 2022).

## Numerical example

The adequacy of our methods was tested with a pedigree-based simulated dataset. A 4-trait model including 2 categorical traits−1 binary, the other one with 3 categories−and 2 continuous ones was simulated for 1.8 million animals spanning 20 overlapping generations without directional selection. The systematic effects in the model were sex and generation, whereas the random effect was the additive genetic effect. The Julia (Bezanson et al. 2017) script employed for the simulation is provided in Supplementary Materials.

Benchmark estimated breeding values from a Gibbs sampler (GIBBS) were calculated as the posterior means of 30,000 samples using gibbsf90+ (Lourenco et al. 2022). NR and EM maximum a posteriori estimates were calculated with blup90iod3 (Lourenco et al. 2022) and compared against GIBBS. In all analyses, variance components and thresholds were assumed to be known. Note that using NR and EM for genomic BLUP (GBLUP) and single-step genomic BLUP (ssGBLUP) (Aguilar et al. 2010) is trivial, since the algorithms apply to any set of relationship matrices, regardless if these are pedigree-based, genomic-based, or other.

**Fig. 1.**
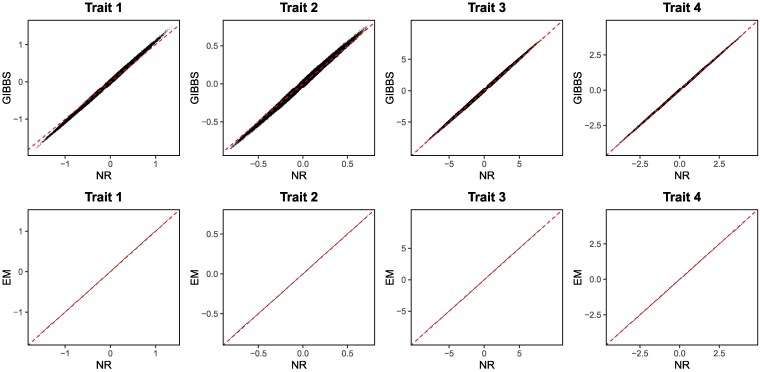
Comparison of estimated breeding values for the simulated dataset. The first 2 traits are categorical, and the others are continuous. The dashed line denotes a line of slope equal to 1 and intercept equal to zero. Estimated breeding values were calculated as posterior means via the Gibbs sampler (GIBBS) or as joint posterior modes computed via Newton-Raphson (NR) or Expectation-Maximization (EM) algorithms.

The agreement between GIBBS, NR, and EM was excellent for all the traits (Fig. 1). NR and EM gave almost identical solutions. The intercept and slope of the regression of GIBBS on NR or EM for the 4 traits ranged 0 to 0.01 and 0.99 to 1.07, respectively. Correlations were always >0.99. Computing times were 3.14 d, 29 min, and 1.1 h for GIBBS, NR, and EM, respectively. The number of iterations until convergence for NR was 7 and for EM was 24. In preliminary analyses with real data, NR was orders of magnitude faster than EM and GIBSS. For instance, we tested our methods in a dataset with 4 traits (2 binary, 2 continuous) and 1.25 million individuals in the pedigree from which 150,000 had available genotypes. The incidences of each binary trait were 91% and 98%, respectively. The systematic effects of the model were sex and mating group, whereas the random effects were contemporary group and the additive genetic effect. The running time for EM was 22 h whereas NR took only 2 h. GIBBS could not handle such amount of data.

**Fig. 2.**
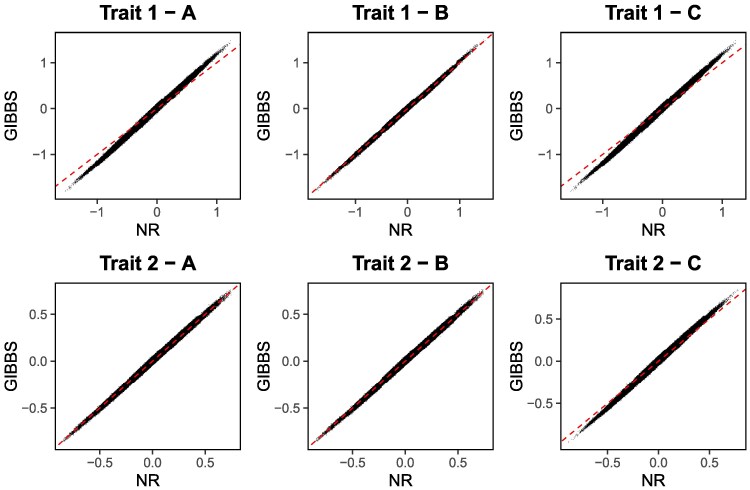
Comparison of estimated breeding values for 3 scenarios with biased additive genetic variances for the categorical trait. Only the 2 categorical traits are shown. The dashed line denotes a line of slope equal to 1 and intercept equal to zero. Estimated breeding values were calculated as posterior means via the Gibbs sampler (GIBBS) or as joint posterior modes computed via the Newton-Raphson (NR) algorithm. The scenarios for biased variance components were: a) (20% reduction for the additive variance in trait 1 and 10% increase for the additive variance in trait 2), b) (20% increase for the additive variance in trait 1 and 10% reduction for the additive variance in trait 2), and c) (20% reduction for the additive variance in both traits).

Because our method assumes that variance components are known, we performed a sensitivity analysis to examine how predictions change when additive genetic variances are biased. We analyzed 3 scenarios of biased variances. In the first scenario, we reduced the additive genetic variance of the binary trait by 20% and increased the additive genetic variance of the second categorical trait by 10%. In the second scenario, the same variances were increased by 20% and decreased by 10%, respectively. Finally, in the third scenario, both variances were reduced by 20%. The remaining parameters were fixed to their simulated values. Predictions with biased variances were calculated by NR and compared with those from GIBBS, which were calculated with the true parameters. As shown in Fig. 2, predictions from NR calculated with biased genetic variances for the categorical traits were still in very good agreement with those from GIBBS. This indicates that small biases in estimated variance components may not alter selection decisions when using EBV calculated by our methods.

## Discussion

Wright's threshold model postulating an unobserved liability is of interest for several reasons, particularly because the existing linear model and quantitative genetics theory apply to the Gaussian liability scale, but not to the observed discrete scale. Therefore, the concepts and algorithms used in the linear scale such as BLUP can be used for threshold traits in the underlying liability scale. Moreover, results can be interpreted in probability terms that are helpful for breeders (Van Tassell et al. 2003; Hidalgo et al. 2024). However, many evaluations of categorical traits fit them as linear traits due to lack of software, a lack of flexibility of the software, or long computing times, especially after genomic information became available. Although flexible and easy to implement, Gibbs sampling is too slow and memory intense for large scale use. This is partly because of extremely slow mixing due to single component updating (Tempelman 1998). In addition, doing a Gibbs sampler single-site update with the mixture of dense and sparse matrices typical of ssGBLUP (Aguilar et al. 2010) is time and memory-consuming.

The MAP estimators initially developed by Gianola and Foulley (1983) are a more appealing option for large-scale applications and genetic evaluations. They define a set of nonlinear equations, which upon convergence provide solutions that are optimal in a statistical sense. The traditionally used option of Fisher-Scoring iteration in these models also estimates thresholds (Hoeschele et al. 1995). However, MAP estimators required quite dedicated programming and moreover, there were so far no existing algorithms for multiple categorical traits.

In this work, we developed 2 completely general algorithms—NR and EM—for the threshold models with multiple categorical and continuous traits. We numerically compared them with its Gibbs sampler counterpart. As expected, computing was faster than with Gibbs sampling and differences in solutions were negligible, therefore validating our algebra and programming. Our algorithms can be fit with any covariance structure for random effects like genomic information, single-step models, correlated effects, etc.

Our Newton-Raphson formulation resembles the method proposed by Höschele et al. (1986) and Foulley et al. (1987). The primary difference between our method and theirs is that ours can account for multiple categorical and continuous traits, whereas theirs only accepts multiple binary traits without any continuous ones. The key methodological difference that allows our generalization is that we express the joint probability in Eq. (17) with a *c*-dimensional integral, while Höschele et al. (1986) and Foulley et al. (1987) express it as a sequence of univariate integrals where one of the bounds was plus or minus infinite (note that for binary traits, both formulations are equal). Based on that difference, we were able to write the first and second derivatives of the hierarchical likelihood in terms of the moments of a multivariate truncated normal distribution. In contrast, Höschele et al. (1986) and Foulley et al. (1987) wrote the first and second derivatives using complicated products of multivariate normal integrals, which are hard to generalize for an arbitrary number of categorical and continuous traits.

Unlike the current state of the art for single categorical and many continuous traits (Hoeschele et al. 1995), replacing the Newton-Raphson iteration with Fisher scoring (ie taking the negative of the expected value of Eq. (26)) does not appear to provide any benefit and may even increase computing time. Moreover, as pointed out by Quaas (1994), the Newton-Raphson formulation can provide, from a frequentist point of view, approximate asymptotic error variances for θ that could be preferable than those obtained with the Fisher information (Efron and Hinkley 1978). In our method, posterior variances, or prediction error variances from a frequentist point of view, can be obtained from the inverse of $\left(W^{\prime }{\tilde{R}}^{-1}W+S\right)$ at convergence. This fact opens several overlooked research topics, like approximation of accuracies of estimated breeding values (Ben Zaabza et al. 2023), and single-step GWAS (Aguilar et al. 2019; Leite et al. 2024) in threshold-linear models. Although the EM formulation does not readily give any approximated variances, there exist simple procedures to obtain it (Louis 1982).

The conceptual framework of this study can be modified to include censored traits into the model (Korsgaard et al. 2003). For that, an extra term in the log-posterior should be added in Eq. (18) and (28). Note that the current terms may remain unchanged due to conditional distribution properties (only conditional variances and expectations may change). For multiple-trait censored models, the added extra term should presumably contain a multivariate normal distribution and a multivariate mass point as the boundary for the traits. This development may require a separate study.

As presented in this study, both the NR and EM algorithms require known variance components and thresholds. At this moment, both parameters need to be estimated by a Bayesian model coupled with a Gibbs sampler and considered as known (fixed) for routine evaluations until there is a re-estimation. For variance components in continuous traits, and for thresholds in some cases, this is a widespread practice in animal breeding (Wang et al. 1997). An alternative approach to estimate variances and thresholds is to consider an adjusted hierarchical likelihood for Eq. (18), as proposed by Lee and Nelder (1996). Although derivatives with respect to thresholds can be obtained from Eq. (18) with the Leibniz integral rule, this may imply a prohibitive number of integrations. Possibly, some refined tools like numerical differentiation or expectation conditional maximization (Meng and Rubin 1993) would be needed for estimating both variance components and thresholds in a future study. Nonetheless, for practical purposes, it seems more straightforward to estimate variance components and thresholds by a Bayesian Gibbs sampler and use them in subsequent evaluations.

## Conclusions

We extended the MAP method for a threshold model to account for multiple categorical and continuous traits. The MAP can be obtained with the Newton-Raphson or Expectation-Maximization algorithms. Both alternatives were orders of magnitude more efficient than the Gibbs sampler threshold-linear model and could be applied in very large data sets. The Newton-Raphson algorithm is faster than the Expectation-Maximization algorithm, and it should be preferred for genetic evaluations. Currently, the methods require known variance components and thresholds, which can be calculated in advance for small data sets using a Gibbs sampler. These developments solved a decades-old methodological gap, enabling accurate, large-scale genetic predictions of categorical traits under multi-trait models, unlocking the full potential of genetic and genomic selection for complex traits in animal and plant populations and genomic-based predictions in clinical genetics.

## Supplementary Material

iyag086_Supplementary_Data

## Data Availability

The script for data simulation is provided as Supplementary Material. Blupf90+ is available for download at https://nce.ads.uga.edu/. Blup90iod3 is currently available only under research agreement with the Animal Breeding and Genetics group at UGA (https://nce.ads.uga.edu/). Supplemental material available at GENETICS online.

## Appendix A

The first partial derivative of each $\log\;P_{i}$ with respect to θ is:

(A1.1)
$$\frac{\partial }{\partial \theta^{\prime }}\log\;(P_{i})=\frac{1}{P_{i}}\int_{T_{k}}\frac{\partial }{\partial \theta^{\prime }}\phi (l_{i};\mu_{i},\Sigma_{i})dl_{i}$$

Applying the chain rule:

(A1.2)
$$\frac{\partial }{\partial \theta^{\prime }}\log\;(P_{i})=\frac{1}{P_{i}}\int_{T_{k}}\phi (l_{i};\mu_{i},\Sigma_{i})\frac{\partial }{\partial \theta^{\prime }}{\mu^{\prime }}_{i}\frac{\partial }{\partial {\mu^{\prime }}_{i}}\left(-\frac{1}{2}{\left(l_{i}-\mu_{i}\right)}^{\prime }\Sigma_{i}^{-1}(l_{i}-\mu_{i})\right)\;dl_{i}$$

Noting that $\frac{\partial }{\partial \theta^{\prime }}{\mu^{\prime }}_{i}={{\tilde{W}}^{\prime }}_{i}$ and $\frac{\partial }{\partial {\mu^{\prime }}_{i}}\left(-\frac{1}{2}{\left(l_{i}-\mu_{i}\right)}^{\prime }\Sigma_{i}^{-1}(l_{i}-\mu_{i})\right)=\Sigma_{i}^{-1}(l_{i}-\mu_{i})$, the derivatives gives:

(A1.3)
$$\frac{\partial }{\partial \theta^{\prime }}\log\;(P_{i})=\frac{1}{P_{i}}{{\tilde{W}}^{\prime }}_{i}\Sigma_{i}^{-1}\int_{T_{k}}(l_{i}-\mu_{i})\phi (l_{i};\mu_{i},\Sigma_{i})dl_{i}$$

where ${{\tilde{W}}^{\prime }}_{i}={\left[\begin{array}{cc} W_{1_{i}} & -R_{12}R_{22}^{-1} \end{array}W_{2_{i}}\right]}^{\prime }$. Now, by definition of the multivariate truncated normal density (Tallis 1961), $\phi_{T}(l_{i};\mu_{i},\Sigma_{i})=\frac{\phi (l_{i};\mu_{i},\Sigma_{i})}{P_{i}}$. Thus, Eq. (A1.3) is:

(A1.4)
$$\frac{\partial }{\partial \theta^{\prime }}\log\;(P_{i})={{\tilde{W}}^{\prime }}_{i}\Sigma_{i}^{-1}\int_{T_{k}}(l_{i}-\mu_{i})\phi_{T}(l_{i};\mu_{i},\Sigma_{i})dl_{i}={{\tilde{W}}^{\prime }}_{i}\Sigma_{i}^{-1}(E_{T}[l_{i}]-\mu_{i})={{\tilde{W}}^{\prime }}_{i}\Delta_{i}$$

where $E_{T}[l_{i}]$ denotes the expectation of $l_{i}$ distributed as a multivariate truncated normal distribution with mean $\mu_{i}$, variance $\Sigma_{i}$, and truncation region $T_{k}$.

## Appendix B

The derivative to find is in Eq. (22):

(A2.1)
$$\begin{array}{rl} \frac{\partial }{\partial \theta }{{\tilde{W}}^{\prime }}_{i}\Delta_{i} & ={{\tilde{W}}^{\prime }}_{i}\frac{\partial }{\partial \theta }E_{T}[\Sigma_{i}^{-1}(l_{i}-\mu_{i})]\\ & ={{\tilde{W}}^{\prime }}_{i}\int_{T_{k}}\frac{\partial }{\partial \theta }\Sigma_{i}^{-1}(l_{i}-\mu_{i})\phi_{T}(l_{i};\mu_{i},\Sigma_{i})dl_{i} \end{array}$$

Applying the product rule to $\Sigma_{i}^{-1}(l_{i}-\mu_{i})\phi_{T}(l_{i};\mu_{i},\Sigma_{i})$:

(A2.2)
$$\frac{\partial }{\partial \theta }{{\tilde{W}}^{\prime }}_{i}\Delta_{i}={{\tilde{W}}^{\prime }}_{i}\left(\int_{T_{k}}\frac{\partial }{\partial \theta }(\Sigma_{i}^{-1}(l_{i}-\mu_{i}))\phi_{T}(l_{i};\mu_{i},\Sigma_{i})+\Sigma_{i}^{-1}(l_{i}-\mu_{i})\frac{\partial }{\partial \theta }\phi_{T}(l_{i};\mu_{i},\Sigma_{i})dl_{i}\right)$$

Splitting the integral and working with the first term:

(A2.3)
$$\int_{T_{k}}\frac{\partial }{\partial \theta }(\Sigma_{i}^{-1}(l_{i}-\mu_{i}))\phi_{T}(l_{i};\mu_{i},\Sigma_{i})dl_{i}=\int_{T_{k}}\frac{\partial }{\partial \mu_{i}}(\Sigma_{i}^{-1}(l_{i}-\mu_{i}))\frac{\partial }{\partial \theta }\mu_{i}\phi_{T}(l_{i};\mu_{i},\Sigma_{i})dl_{i}$$

which gives:

(A2.4)
$$\int_{T_{k}}\frac{\partial }{\partial \theta }(\Sigma_{i}^{-1}(l_{i}-\mu_{i}))\phi_{T}(l_{i};\mu_{i},\Sigma_{i})dl_{i}\;=-\Sigma_{i}^{-1}{\tilde{W}}_{i}\int_{T_{k}}\phi_{T}(l_{i};\mu_{i},\Sigma_{i})dl_{i}=-\Sigma_{i}^{-1}{\tilde{W}}_{i}$$

For the second term of the integral in Eq. (A2.2), note that the quotient rule must be applied because $\phi_{T}(l_{i};\mu_{i},\Sigma_{i})=\frac{\phi (l_{i};\mu_{i},\Sigma_{i})}{P_{i}}$. Thus:

(A2.5)
$$\begin{array}{rl} & \int_{T_{k}}\Sigma_{i}^{-1}(l_{i}-\mu_{i})\frac{\partial }{\partial \theta }\phi_{T}(l_{i};\mu_{i},\Sigma_{i})dl_{i}\\ & \;=\int_{T_{k}}\Sigma_{i}^{-1}(l_{i}-\mu_{i})\left(\frac{\partial }{\partial \theta }\phi (l_{i};\mu_{i},\Sigma_{i})\frac{1}{P_{i}}+\phi (l_{i};\mu_{i},\Sigma_{i})\frac{\partial }{\partial \theta }\left(\frac{1}{P_{i}}\right)\right)dl_{i} \end{array}$$

For the first term of the addend, note that from Appendix 1, $\frac{\partial }{\partial \theta }\phi (l_{i};\mu_{i},\Sigma_{i})=\phi (l_{i};\mu_{i},\Sigma_{i})(l_{i}-\mu_{i}{)}^{\prime }\Sigma_{i}^{-1}{\tilde{W}}_{i}$. Thus:

(A2.6)
$$\begin{array}{rl} & \int_{T_{k}}\Sigma_{i}^{-1}(l_{i}-\mu_{i})\frac{1}{P_{i}}\frac{\partial }{\partial \theta }\phi (l_{i};\mu_{i},\Sigma_{i})dl_{i}\\ & \;=\;\int_{T_{k}}\Sigma_{i}^{-1}(l_{i}-\mu_{i}){\left(l_{i}-\mu_{i}\right)}^{\prime }\Sigma_{i}^{-1}\phi_{T}(l_{i};\mu_{i},\Sigma_{i})dl_{i}{\tilde{W}}_{i}\\ & \;=\;E_{T}[\Sigma_{i}^{-1}(l_{i}-\mu_{i}){\left(l_{i}-\mu_{i}\right)}^{\prime }\Sigma_{i}^{-1}]{\tilde{W}}_{i} \end{array}$$

For the second term of Eq. (A2.5), just apply the chain rule and Appendix 1, giving $\frac{\partial }{\partial \theta }\left(\frac{1}{P_{i}}\right)=\frac{-1}{P_{i}^{2}}\int_{T_{k}}{\left(l_{i}-\mu_{i}\right)}^{\prime }\Sigma_{i}^{-1}{\tilde{W}}_{i}\phi (l_{i};\mu_{i},\Sigma_{i})dl_{i}$. Then:

(A2.7)
$$\begin{array}{rl} & \int_{T_{k}}\Sigma_{i}^{-1}(l_{i}-\mu_{i})\phi (l_{i};\mu_{i},\Sigma_{i})dl_{i}\frac{\partial }{\partial \theta }\left(\frac{1}{P_{i}}\right)\\ & \;=-\int_{T_{k}}\Sigma_{i}^{-1}(l_{i}-\mu_{i})\phi_{T}(l_{i};\mu_{i},\Sigma_{i})dl_{i}\int_{T_{k}}{\left(l_{i}-\mu_{i}\right)}^{\prime }\Sigma_{i}^{-1}\phi_{T}(l_{i};\mu_{i},\Sigma_{i})dl_{i}{\tilde{W}}_{i}\\ & \;=E_{T}[\Sigma_{i}^{-1}(l_{i}-\mu_{i})]E_{T}[{\left(l_{i}-\mu_{i}\right)}^{\prime }\Sigma_{i}^{-1}]{\tilde{W}}_{i} \end{array}$$

Merging the results from Eq. (A2.6 to A2.7) into Eq. (A2.5):

(A2.8)
$$\int_{T_{k}}\Sigma_{i}^{-1}(l_{i}-\mu_{i})\frac{\partial }{\partial \theta }\phi_{T}(l_{i};\mu_{i},\Sigma_{i})dl_{i}=(E_{T}[\Sigma_{i}^{-1}(l_{i}-\mu_{i}){\left(l_{i}-\mu_{i}\right)}^{\prime }\Sigma_{i}^{-1}]-E_{T}[\Sigma_{i}^{-1}(l_{i}-\mu_{i})]E_{T}[{\left(l_{i}-\mu_{i}\right)}^{\prime }\Sigma_{i}^{-1}]){\tilde{W}}_{i}=\mathrm{Va}r_{T}(\Sigma_{i}^{-1}(l_{i}-\mu_{i})){\tilde{W}}_{i}=\Sigma_{i}^{-1}\mathrm{Va}r_{T}(l_{i})\Sigma_{i}^{-1}{\tilde{W}}_{i}$$

where $\mathrm{Va}r_{T}(l_{i})$ denotes the variance of $l_{i}$ distributed as a truncated multivariate normal vector with mean $\mu_{i}$, variance $\Sigma_{i}$, and truncation region $T_{k}$. Note that $\mathrm{Va}r_{T}(l_{i})$ has no closed-form expression.

Finally, combining Eq. (A2.4) and Eq. (A2.8) in Eq. (A2.2):

(A2.9)
$$\frac{\partial }{\partial \theta }{{\tilde{W}}^{\prime }}_{i}\Delta_{i}={{\tilde{W}}^{\prime }}_{i}(-\Sigma_{i}^{-1}{\tilde{W}}_{i}+\Sigma_{i}^{-1}\mathrm{Va}r_{T}(l_{i})\Sigma_{i}^{-1}{\tilde{W}}_{i})=-{{\tilde{W}}^{\prime }}_{i}(\Sigma_{i}^{-1}-\Sigma_{i}^{-1}\mathrm{Va}r_{T}(l_{i})\Sigma_{i}^{-1}){\tilde{W}}_{i}=-{{\tilde{W}}^{\prime }}_{i}\Gamma_{i}{\tilde{W}}_{i}$$
